# Islet Autoantibody Patterns in Patients With Type 2 Diabetes Aged 60 and Higher: A Cross-Sectional Study in a Chinese Hospital

**DOI:** 10.3389/fendo.2018.00260

**Published:** 2018-05-25

**Authors:** Rumei Li, Jinya Huang, Yifei Yu, Yehong Yang

**Affiliations:** Department of Endocrinology, Huashan Hospital, Fudan University, Shanghai, China

**Keywords:** islet autoantibody, insulin autoantibodies, islet cell cytoplasmic autoantibodies, glutamic acid decarboxylase autoantibodies, type 2 diabetes, elderly

## Abstract

**Background:**

Some elderly citizens with a clinical diagnosis of type 2 diabetes had evidence of positive islet autoantibodies. We aimed to discover their islet autoantibody patterns and independent correlative factors that might lead to a better understanding of significance of islet autoimmunity in the progression of elderly diabetes.

**Methods:**

A total of 541 inpatients of clinically diagnosed type 2 diabetes aged 60 and over were recruited. Three islet autoantibodies including insulin autoantibody (IAA), islet cell antibody (ICA), and glutamic acid decarboxylase antibody (GADA) as well as clinical and biochemical characteristics were tested and collected in Huashan Hospital. Associations between these antibodies and clinical features were analyzed by Spearman correlation and binary logistic analyses.

**Results:**

In our current study, total positive rate of islet autoantibodies (IAA, ICA, and GADA) was 35.67% with 26.62% for individual IAA, 5.55% for ICA, and 5.91% for GADA, in elderly with type 2 diabetes. None of combinations of such autoantibodies were observed, with the exception of IAA + ICA (0.74%, *n* = 4), IAA + GADA (1.48%, *n* = 8), and ICA + GADA (0.18%, *n* = 1). Compared with GADA negative group, patients in positive group tended to have lower level of fasting and postprandial C peptide, fasting blood glucose (FBG), and body mass index (BMI). After adjusted for the BMI, FBG, and postprandial C peptide, fasting C peptide seemed to be an independent factor related to GADA positivity (OR = 0.52, *p* = 0.02). As for patients with positive IAA, they were more likely to have insulin treatment with longer duration of diabetes, higher level of BMI, and lower level of postprandial C peptide. After adjusted for the duration of diabetes, BMI, and postprandial C peptide, insulin treatment was a significant predictor for IAA positivity (OR = 5.20, *p* < 0.0001). Furthermore, hs-CRP was positively related to ICA positivity, and hs-CRP appeared to be an independent indicator for ICA (OR = 3.43, *p* = 0.008).

**Conclusion:**

In elderly with type 2 diabetes, high prevalence rate of IAA was frequently accompanied with insulin treatment, while ICA and GADA were more closely associated with the systemic inflammation and beta-cell failure, respectively.

## Introduction

Historically, islet autoimmunity was the hallmark of classical type 1 diabetes, which distinguishes it from type 2 diabetes. Signs of autoimmune abnormalities such as insulin autoantibody (IAA), islet cell antibody (ICA), and glutamic acid decarboxylase antibody (GADA) predicted rapid progression to insulin-requiring diabetes (1). However, accumulating evidences (2–4) had substantiated that a group of adult patients clinically diagnosed with type 2 diabetes exhibited positivity of autoantibodies as well. The condition was often termed latent autoimmune diabetes in adults (LADA). LADA was viewed as type 1.5 diabetes because of a combination of features of both type 2 diabetes, such as late-onset and non-insulin-requiring at diagnosis, and type 1 diabetes with positivity in islet autoantibodies (5). Overall, the prevalence of islet autoantibodies was 52–74% in type 1 diabetes, while about 5% in type 2 diabetes and 1% in healthy volunteers (6, 7).

However, great challenges were posed to the classification of subtypes of diabetes with the raise of metabolic inflammatory pathogenesis (8) in type 2 diabetes. Currently, solid evidences indicated that overlap exists among various types of diabetes (2). On one hand, systemic inflammation played a role in pathogenesis of type 1, LADA, and type 2 diabetes evidenced by elevated levels of cytokines including interleukin-6, lipocalin 2, high-sensitivity C-reactive protein (hs-CRP), and adiponectin (9). On the other hand, multiple findings supported that B cells promoted the process of type 2 diabetes by supporting T cell-mediated inflammation and thus insulin resistance in both *vitro* and *vivo* (10–12). It was believed that islet autoimmune development in type 2 diabetes was resulted from chronic systemic inflammation (13). More importantly, there was compelling evidence that 23 of the 36 phenotypic type 2 diabetic patients were detected positive T-cell proliferative responses to islet proteins, among whom, 12 patients were dual positive in autoantibody and T cell responses (12). Nevertheless, whether adaptive immune system could generate autoimmune responses in type 2 diabetes as disease progressed is still under research (14, 15).

It was reported that, in type 2 diabetic patients 45 years or younger at onset, the frequency of GADA was 19.3%, while it was 8.2% after 45 years old at onset (16). Besides, during onset age between 55 and 65 years old, the frequency of GADA was approximately 6% (17). But, there was rare information about other autoantibodies such as IAA and ICA in elderly patients presenting with type 2 diabetes. In an effort to assess the islet autoantibodies patterns in elderly type 2 diabetes, we performed such hospital-based cross-sectional study with 541 clinically diagnosed type 2 diabetes inpatients and identified underling correlative factors in the positivity of IAA, ICA, and GADA. Thus, we could have a better understanding about the significance of islet autoantibodies in elderly type 2 diabetes, which might guide our treatment with disease progression.

## Materials and Methods

### Study Population

In the present study, a total of 541 patients were recruited who met the eligibility criteria as follows: (i) clinically diagnosed as type 2 diabetes based on the criteria of the Chinese Diabetes Society and (ii) aged ≥60 years. All the participants were inpatients, who were admitted to the Department of Endocrinology in Huashan Hospital between January 2014 and September 2017. Those who caught fever or were under acute inflammatory status such as obvious urinary infection and respiratory infection were excluded. Islet autoantibodies (IAA, ICA, and GADA) were determined among all the patients. According to the islet autoantibodies status, participants were divided into groups including IAA negative and positive group (*n* = 397/144), ICA negative and positive group (*n* = 511/30), and GADA negative and positive group (*n* = 509/32). Clinical and biochemical characteristics were evaluated and compared among different groups. The protocol was approved by the institutional review board of Basic Medical College of Fudan University (2016-Y028). Written informed consent was obtained from all participants in accordance with the Declaration of Helsinki.

### Antibody Measurements

Serum samples were stored at 4°C and analyzed within 24 h. In brief, IAA, ICA, and GADA were examined centrally by Department of Clinical Laboratory in Huashan Hospital (Shanghai, China). IAA and ICA were qualitatively determined by ELISA kits (Biomerica, USA). All samples were measured in duplicate. We detected the absorbance of each pore at the wavelength of 405 nm in ELISA meter and calculated the average optical density value for reference control (*R_a_*), negative control (*N_a_*), positive control (*P_a_*), which were contained in ELISA kit, and samples (*S_a_*). The results were expressed in an index (index = *S_a_*/*R_a_*). The cut-off point for a positive sample in these ELISA kits was set at >1.05. Meanwhile, as quality control, *N_a_*/*R_a_* < 0.95 and *P_a_*/*R_a_* > 1.05 proved the validity of experiment.

Glutamic acid decarboxylase antibody was quantitatively determined by ELISA kit (Euroimmun AG, Germany) according to the manufacturer’s instructions. Positivity of GADA was defined by 5 IU/ml or higher. The sensitivity and specificity of GADA were 92% and 98%, respectively, as evaluated in the Diabetes Autoantibody Standardization Program (DASP 2003).

### Data Collection

The demographic and metabolic features were collected *via* the history system in hospital, including age, gender (male/female), duration of diabetes, current or history of insulin treatment (yes/no), fasting blood glucose (FBG), hemoglobin A1c (HbA1c), fasting C peptide, postprandial C peptide, as well as number of patients with higher level of high-sensitive C-reactive protein (hs-CRP ≥ 3.25 mg/l). Body mass index (BMI) was calculated as weight/height^2^ (kg/m^2^).

### Statistical Analysis

Numerical variables and categorical variables were presented as mean ± standard deviation (SD) and number (percentage) of patients, respectively. Independent samples *t*-test for parametric data and the Mann–Whitney *U*-test for non-parametric data while chi-square test for numbers and percentages were performed to compare the differences between two independent groups. The results of GADA were translated into negative and positive with the cut point of 5 IU/ml. The relationships between islet autoantibodies and clinical features were calculated by Spearman’s rank correlation coefficient. Binary logistic regression analysis was also used to confirm the independent factors relative with autoantibodies positivity after adjusted for possible confounding variables. A two-sided *p*-value of <0.05 was considered as statistical significance. All the data were analyzed using SPSS version 18 (IBM, USA).

## Results

### Rare Combination of Islet Autoantibodies Observed in Elderly Type 2 Diabetes

A total of 541 patients with clinically diagnosed type 2 diabetes were recruited, aged from 60 to 95 years, with 280 males (51.76%) and 261 females (48.24%). Among all the elderly with type 2 diabetes in this analysis, 35.67% (*n* = 193) of participants were detected with at least one positive islet autoantibody among these three autoantibodies (Table 1). As showed in Table 1, the individual prevalence rate of three islet autoantibodies was 26.62% for IAA (*n* = 144), 5.55% for ICA (*n* = 30), and 5.91% for GADA (*n* = 32). There was rare combination of different autoantibodies observed in elderly diabetes patients, apart from IAA + ICA (0.74%, *n* = 4), IAA + GADA (1.48%, *n* = 8), and ICA + GADA (0.18%, *n* = 1). None simultaneously positive of three antibodies were observed in type 2 diabetes aged 60 and over.

**Table 1:** Detection of serum autoantibodies and their combinations in elderly type 2 diabetes.

Autoantibody or its combination	Positive numbers	Positive rate (%)
IAA	144	26.62
ICA	30	5.55
GADA	32	5.91
IAA + ICA	4	0.74
IAA + GADA	8	1.48
ICA + GADA	1	0.18
IAA + ICA + GADA	0	0
IAA/ICA/GADA	193	35.67

*“+” means simultaneously positive, “/” means at least one positive, the first three rows were positive for individual antibody*.

*IAA, insulin autoantibodies; ICA, islet cell cytoplasmic autoantibodies; GADA, glutamic acid decarboxylase autoantibodies*.

### Clinical and Biochemical Characteristics of Islet Autoantibody Positive Subjects in Elderly Type 2 Diabetes

The demographic and clinical characteristics of the participants were presented according to the positivity of islet autoantibodies (Table 2). Compared with IAA negative group, IAA positive group tended to have longer duration of diabetes, higher level of BMI, but lower level of postprandial C peptide (*p* < 0.05). Most significantly, about 85.40% of IAA positive patients had history of insulin treatment, much higher than that of IAA negative patients (52.40%, *p* < 0.05). There was no significant difference in the distribution of age, gender, duration of diabetes, BMI, FBG, HbA1c, fasting, and postprandial C peptide and history of insulin treatment between ICA negative and positive groups except for the percentage of higher level of serum hs-CRP. About 50% of those with positive ICA showed higher level of hs-CRP while about 22.60% of ICA negative patients had higher level of hs-CRP. As presented in Table 2, the plasma level of FBG, fasting C peptide, and BMI were significantly lower in GADA positive participants than those in GADA negative groups (*p* < 0.05). Moreover, the level of postprandial C peptide seemed slightly lower in GADA positive groups than that of GADA negative group (*p* = 0.08).

**Table 2:** Clinical and biochemical characteristics of each group of subjects in type 2 diabetes aged 60 and over.

	IAA negative	IAA positive	*p* Value	ICA negative	ICA positive	*p* Value	GADA Negative	GADA Positive	*p* Value
Number of patients	397 (73.38%)	144 (26.62%)	/	511 (94.45%)	30 (5.55%)	/	509 (94.09%)	32 (5.91%)	/
Age (years)	69.65 ± 7.86	68.72 ± 7.09	0.30	69.42 ± 7.69	69.07 ± 7.45	0.88	69.50 ± 7.70	67.72 ± 7.02	0.18
Gender (male/female)	204/193 (51.40/48.60%)	76/68 (52.80/47.20%)	0.78	265/246 (51.90/48.10%)	15/15 (50.00/50.00%)	0.84	263/246 (51.70/48.30%)	17/15 (53.10/46.90%)	0.87
Duration of diabetes (years)	11.89 ± 8.05	14.38 ± 7.72	0.001	12.55 ± 8.04	12.57 ± 8.04	0.93	12.71 ± 8.02	10.10 ± 7.88	0.11
BMI (kg/m^2^)	24.38 ± 3.59	25.26 ± 3.42	0.005	24.63 ± 3.56	24.42 ± 3.73	0.91	24.71 ± 3.57	23.20 ± 3.20	0.01
FBG (mmol/l)	8.22 ± 3.75	8.03 ± 3.67	0.43	8.21 ± 3.77	7.46 ± 2.85	0.44	8.22 ± 3.70	7.32 ± 4.04	0.02
HbA1c (%)	8.45 ± 2.01	8.37 ± 1.71	0.96	8.46 ± 1.95	7.95 ± 1.63	0.20	8.44 ± 1.94	8.21 ± 1.76	0.57
Fasting C peptide (μg/l)	1.43 ± 1.07	1.29 ± 0.94	0.16	1.39 ± 1.05	1.41 ± 0.98	0.67	1.42 ± 1.05	0.96 ± 0.88	0.006
postprandial C peptide (μg/l)	3.95 ± 2.82	3.35 ± 2.75	0.02	3.76 ± 2.80	4.31 ± 3.02	0.26	3.83 ± 2.80	3.08 ± 3.02	0.08
hs-CRP ≥ 3.25 mg/l	72 (25.00%)	21 (21.00%)	0.42	83 (22.60%)	10 (50.00%)	0.005	89 (24.10%)	4 (21.10%)	0.98
Insulin treatment	208 (52.40%)	123 (85.40%)	<0.0001	313 (61.30%)	18 (60.00%)	0.89	309 (60.70%)	22 (68.80%)	0.37

*Patients were classified with regard to the positivity of islet autoantibodies (IAA, ICA, GADA). Data are shown as mean ± SD and *n* (%), unless otherwise indicated*.

*p Values refer to the comparison of the two groups by independent samples t-test, the Mann–Whitney U-test or chi-square test*.

*A p-value of <0.05 was considered statistical significance*.

*BMI, body mass index; FBG, fasting blood glucose; HbA1c, hemoglobin A1c; hs-CRP, high-sensitive C-reactive protein*.

### Correlation Analysis of Islet Autoantibodies in Elderly Type 2 Diabetes

In order to explore in underling factors that might play an important role in the positivity of islet autoantibodies in elderly type 2 diabetes, we calculated the correlation coefficient between individual islet autoantibody and clinical features. Event of insulin treatment was strongly associated with positivity of IAA (*r* = 0.299, *p* < 0.0001), whereas, the level of postprandial C peptide (*r* = −0.103, *p* = 0.02) was negatively related to it. Besides, duration of diabetes (*r* = 0.144, *p* = 0.001) and BMI of patients (*r* = 0.102, *p* = 0.019) were also indicators for the positivity of IAA in elderly diabetes. As for the ICA, there seemed to be only one positive determinant, hs-CRP (*r* = 0.142, *p* = 0.005), though it was not associated with other autoantibodies such as IAA and GADA. Moreover, serum level of both fasting and postprandial C peptide appeared to be significantly negatively correlated to GADA (*r* = −0.120, *p* = 0.005; *r* = −0.077, *p* = 0.081, respectively). In addition, BMI (*r* = −1.019, *p* = 0.011) and FBG (*r* = −0.097, *p* = 0.024) were negatively related with GADA (Table 3). According to the results of correlation analysis of islet autoantibodies, binary logistic analysis was performed to confirm the independent factors that related with antibody positivity. As the results indicated, insulin treatment was a significant positive predictor of IAA (OR = 5.20, 95%CI 2.96–9.14, *p* < 0.0001) after adjusted for BMI, duration of diabetes, and postprandial C peptide, while level of fasting C peptide was a significant negative predictor of GADA (OR = 0.52, 95%CI 0.30–0.91, *p* = 0.02) with correction for BMI, FBG, and postprandial C peptide. Among all the clinical and biochemical variables collected in this analysis, higher levels of hs-CRP showed the only association with ICA (OR = 3.43, 95%CI 1.38–8.53, *p* = 0.008) (Table 4).

**Table 3:** Correlation analysis of islet autoantibodies in elderly type 2 diabetes.

	IAA (*n* = 144)	ICA (*n* = 30)	GADA (*n* = 32)
Age (years)			
*r*	−0.044	−0.007	−0.058
*p*	0.304	0.876	0.179
Gender (male/female)			
*r*	−0.012	0.009	−0.007
*p*	0.775	0.843	0.873
Duration of diabetes (years)			
*r*	**0.144**	0.004	−0.070
*p*	**0.001**	0.930	0.105
BMI (kg/m^2^)			
*r*	**0.102**	−0.005	−**0.109**
*p*	**0.019**	0.914	**0.011**
HbA1c (%)			
*r*	0.002	−0.055	−0.025
*p*	0.963	0.203	0.566
Fasting blood glucose (mmol/l)			
*r*	−0.034	−0.034	−**0.097**
*p*	0.427	0.438	**0.024**
Fasting C peptide (μg/l)			
*r*	−0.061	0.018	−**0.120**
*p*	0.161	0.670	**0.005**
postprandial C peptide (μg/l)			
*r*	−**0.103**	0.050	−0.077
*p*	**0.020**	0.264	0.081
hs-CRP ≥ 3.25 (mg/l)			
*r*	−0.038	**0.142**	−0.042
*p*	0.461	**0.005**	0.406
Insulin treatment			
*r*	**0.299**	−0.006	0.039
*p*	**<0.0001**	0.891	0.366

*Shown are r and p values from Spearman analysis. A p-value of <0.05 was considered as statistical significance. Significant correlations are in bold*.

*IAA, insulin autoantibodies; ICA, islet cell cytoplasmic autoantibodies; GADA, glutamic acid decarboxylase autoantibodies; BMI, body mass index; HbA1c, hemoglobin A1c; hs-CRP, high-sensitive C-reactive protein*.

**Table 4:** Binary logistic analysis of islet autoantibodies in elderly type 2 diabetes.

	OR (95%CI)	*p* Value
**IAA**		
Insulin treatment^a^	5.20 (2.96–9.14)	<0.0001
BMI (kg/m^2^)	1.34 (0.95–1.88)	0.10
Duration of diabetes (years)	1.01 (0.98–1.04)	0.48
postprandial C peptide (μg/l)	0.99 (0.91–1.08)	0.91
**ICA**		
hs-CRP ≥ 3.25 mg/l	3.43 (1.38–8.53)	0.008
**GADA**		
Fasting C peptide (μg/l)^b^	0.52 (0.30–0.91)	0.02
postprandial C peptide (μg/l)	1.03 (0.83–1.29)	0.78
BMI (kg/m^2^)	0.57 (0.26–1.22)	0.14
FBG (mmol/l)	0.94 (0.83–1.06)	0.31

*p Value was calculated from binary logistic regression*.

*^a^Adjusted for BMI, duration of diabetes, and postprandial C peptide based on the results of correlation analysis of IAA*.

*^b^Adjusted for BMI, fasting blood glucose, and postprandial C peptide based on the results of correlation analysis of GADA*.

*IAA, insulin autoantibodies; ICA, islet cell cytoplasmic autoantibodies; GADA, glutamic acid decarboxylase autoantibodies, BMI, body mass index; hs-CRP, high-sensitive C-reactive protein; FBG, fasting blood glucose; OR, odds ratio; CI, confidence interval*.

## Discussion

Previous studies had indicated that a wide range of 4–17% of apparent type 2 diabetes had markers of islet autoimmunity as seen in type 1 diabetes (6, 18, 19). This was not surprising because innate as well as adaptive immune system might have a role in all types of diabetes, including type 2 diabetes (8, 20, 21). In the current study, the participants were aged 60 and over, with average duration of diabetes about 12 years, indicating that the onset of diabetes tended to happen in their late forties and even early fifties. Our study showed that approximately 5.6 and 5.9% elderly T2D patients were positive for ICA and GADA, respectively, similar to previous Chinese study (22) and UK study (23), but there was a relatively higher frequency of GADA than that in Japanese study (3.8%) (24). Other islet autoantibodies, namely insulinoma-associated protein-2 (IA-2) (25) and zinc transporter 8 (ZnT8) (26), were reported to be found in only 2.2 and 1.8% of patients with T2D. However, according to our research, the majority of elderly type 2 diabetes had a single islet autoantibody. Rare combinations of autoantibodies were found in elderly diabetes. One of the reasons might be that the prevalence of islet autoantibodies was declined with increasing age at diagnosis (23). Besides, a recent study (18) reported that doubled antibody positivity (IA-2 + GADA) was less frequently seen in Asian participants than that in white European participants (1.6 vs 5.7%, *p* < 0.05). However, different laboratory assays for islet autoantibodies might have also affected the outcomes.

In contrast to previous studies (23, 27), our data showed a much higher prevalence rate of IAA (*n* = 144, 26.62%) in elderly type 2 diabetes. It was noteworthy to acknowledge that almost 85.40% of participants were currently or previously treated with insulin injection in IAA positive group, which was found to be positively associated with IAA in the following correlation and logistic analysis (OR: 5.20, *p* < 0.05). Those with IAA positivity were tended to have longer duration of diabetes, higher BMI, and lower level of postprandial C peptide (*p* < 0.05). Therefore, it was reasonable to believe that elderly diabetes with more than 10 years’ duration had a higher tendency to be treated with insulin because of the progression of disease itself, which consequently might result in higher positivity of IAA. Thus, with false impression of exogenous-stimulated IAA, IAA was not a good candidate screened for islet autoimmunity in elderly diabetes (28, 29).

In line with the findings of Prof. Schloot in younger diabetes aged 30–70 (30), GADA positive group had lower BMI than negative group (*p* < 0.05) in elderly diabetes aged over 60. It was also reported (19) that GADA showed decreasing frequencies with increasing BMI in type 2 diabetes. Besides, our study found that level of FBG seemed to be lower in GADA positive group than that in negative group. But, HbA1c had no significant differences between these groups. After adjusted for BMI, fasting and postprandial C peptide, the odd ratio of FBG for positivity of GADA was meaningless (*p* > 0.05). Therefore, the relationship between FBG and GADA might depend on other factors. There were neither significant differences of age, sex, duration of diabetes, nor hs-CRP or insulin treatment between GADA positive and negative group observed in elderly diabetes.

To investigate the underling factors that might have a relationship with the positivity of islet autoantibodies in elderly diabetes, we analyzed the correlation between metabolic features and individual autoantibody. As expected (31), GADA was positively associated with decreased release of both fasting and postprandial C peptides, suggesting beta-cells damage. As previously evidenced by pathology data from type 2 diabetic donors with islet autoimmunity, abating beta-cell mass was observed to be seemingly determined by an immune-mediated injury of pancreatic beta-cells (4). Interestingly, glucose clamp studies indicated that peripheral insulin resistance was presented similarly in both autoantibody positive and negative patients of clinically diagnosed type 2 diabetes (4). These results pointed toward a complex relationship between islet autoimmunity and diabetes types, supporting that islet autoimmunity might work in the progression of type 2 diabetes (32). Other study (23) had suggested that the presence of islet autoantibodies in older age-group diabetes was a weaker predictor of insulin requirement than that in younger group (17). Nevertheless, since the presence of GADA was positively related to short supply of C peptide, illustrating dysfunction and mass loss of beta-cells in pancreas, it was still a potential indicator of need for insulin therapy. However, in our analysis, both correlative and logistic analyses found no relationship between GADA and insulin treatment in these patients, which might be explained by inappropriate use of insulin and poor compliance of patients in China.

Unlike GADA, ICA was only independently related to serum level of hs-CRP (*p* < 0.05), with no significant association with sex, duration of diabetes, BMI, HbA1c, FBG, insulin treatment, fasting, and postprandial C peptide. Hs-CRP, an acute responsive inflammatory protein, indicated the activation of immune system. Interestingly, in contrast to our analysis, ICA was reported to be negatively correlated with TNF-a (*p* = 0.011) (30). The different changes of immune effectors might suggest different pathogenetic mechanisms and feedbacks involved in diabetes. However, hs-CRP has a longer half-life than TNF-a, with greater stability (33) over prolonged periods, and thus, hs-CRP was a better measure of systemic inflammation than other circulating cytokines (34). Since increased BMI was positively associated with higher systemic cytokine concentrations in diabetes (20), ICA positive and negative group shared similar BMI, which got rid of the effect of BMI on hs-CRP. Given that hs-CRP was positively correlated with ICA in elderly type 2 diabetes, systemic inflammation might be responsible for production of ICA in the progression of diabetes.

We analyzed the islet autoantibodies patterns and their independent correlative factors in clinically diagnosed type 2 diabetes at the age of 60 and over with the assumption that it abated the ambiguity for definition of classical type 1 and 2 diabetes. There were some clues that islet autoimmunity also participates in the progression of type 2 diabetes. But, conclusion of this analysis is supposed to be restricted in elderly diabetes. With a small group of participants, an extended group of patients are needed to provide firm evidence. Furthermore, to investigate the changes of islet autoantibodies through time, as well as mutual changes in the serum C peptide and inflammatory cytokines, we have to better carry out a large prospective study in the future to monitor the fluctuation of islet autoantibodies including ICA, GADA, IA-2, ZnT8, and figure out their regulators.

To conclude, it is the first time that we identified the independent positive relationship between serum level of ICA and hs-CRP in elderly diabetes, which supported the connection between ICA and systemic inflammation in elderly diabetes. In line with previous findings, patients with positive GADA had apparent beta-cell failure evidenced by lower serum level of C peptide before or after meal, calling for treatment of insulin. In addition, frequent treatment of insulin was supposed to be an underlying factor stimulating the immune system, leading to such high prevalence of IAA. Herein, we should realize the significance of islet autoantibodies in clinically diagnosed type 2 diabetes and classify the subtypes of diabetes critically. Further study with better understanding of mechanisms of islet autoantibodies might help establish effective methods for intervention and prediction in the future.

## Ethics Statement

The protocol was approved by the institutional review board of Basic Medical College of Fudan University (2016-Y028). Written informed consent was obtained from all participants in accordance with the Declaration of Helsinki.

## Availability of Data and Material

The datasets analyzed during the current study are available from the corresponding author on reasonable request.

## Author Contributions

RL analyzed the data, interpreted the results and was a major contributor in writing the manuscript, while YhY contributed to the conception and design of the study, interpreted results of the data analysis, and provided critical review of the manuscript. JH and YfY collected the data, analyzed the data, and helped write the manuscript. All authors read and approved the final manuscript.

## Conflict of Interest Statement

The authors declare that the research was conducted in the absence of any commercial or financial relationships that could be construed as a potential conflict of interest.
